# Efficacy of *Polygonatum sibiricum* on Mild Insomnia: A Randomized Placebo-Controlled Trial

**DOI:** 10.3390/nu11081719

**Published:** 2019-07-25

**Authors:** Eunji Ha, Haejin Hong, Tammy D. Kim, Gahae Hong, Suji Lee, Seunghee Kim, Nayeon Kim, Sang Duck Jeon, Chang-Won Ahn, Hun Jung Kim, Young-Jin Lee, Sujung Yoon, Geon Ha Kim, Jungyoon Kim

**Affiliations:** 1 Department of Brain and Cognitive Sciences, Ewha Womans University, Seoul 03760, Korea; 2 Ewha Brain Institute, Ewha Womans University, Seoul 03760, Korea; 3 Nongshim Corporation, Seoul 07057, Korea; 4 Department of Neurology, Ewha Womans University College of Medicine, Ewha Womans University Mokdong Hospital, Seoul 07985, Korea

**Keywords:** *Polygonatum*, insomnia, randomized controlled trial, cerebral perfusion, arterial spin labeling, medial prefrontal cortex, default mode network

## Abstract

*Polygonatum sibiricum* (PS) rhizome, which contains glyceryl-1-monolinoleate as its primary active component, has been shown to improve insomnia in animal models. Based on these findings, we aimed to investigate the safety and efficacy of PS rhizome extract in improving sleep quality in individuals with mild insomnia. Eighty individuals with mild insomnia were enrolled in a four-week, randomized, double-blind, placebo-controlled trial of PS rhizome extract (500 mg/day, *n* = 40, PS group) or placebo (*n* = 40, placebo group). The primary outcome measure was change in total score on the Athens Insomnia Scale (AIS) to indicate sleep quality. The secondary outcome measures included change in actigraphy data and perfusion levels in the brain regions within the default mode network (DMN), which is known to play a key role in insomnia. The PS group showed greater improvement in the total AIS score with a significant increase in total sleep time, relative to the placebo group. In addition, significant group-by-visit interactions were observed in the perfusion level of the medial prefrontal cortex within the DMN. Findings of the current study provide first evidence that PS rhizome extract could be an effective natural ingredient for improving sleep in mild insomnia using a human model.

## 1. Introduction

Insomnia is highly prevalent in the general population and is associated with several functional impairments such as increased work absenteeism, higher rates of occupational or transportation-related accidents, and lower quality of life [1,2,3]. In addition, debilitating health conditions including cognitive impairment, emotional disturbance, and increased risks of cardiovascular disease or psychiatric disorders have been reported to be associated with persistent insomnia [4,5,6]. As such, early treatment for sleep disturbances may be warranted to reduce the long-term consequences of insomnia.

The current clinical practice guidelines for pharmacological treatment of insomnia recommends the use of sedative hypnotics including benzodiazepines and benzodiazepine receptor agonists as effective prescription medications for insomnia, rather than any over-the-counter preparations [7]. However, individuals with insomnia are often reluctant to take prescription medications, resulting in low rates of seeking treatments [8,9], partly due to the concerns on potential adverse effects of hypnotics including deterioration of daytime performance, memory loss, drug resistance, and addiction [10]. In contrast, individuals with insomnia are reported to prefer self-help strategies over professional consultations [11]. These self-help strategies often entail natural remedies including herbs, vitamins, and dietary supplements [12].

*Polygonatum sibiricum* (PS), which is a flowering plant belonging to the family Liliaceae, is one of the natural products commonly ingested as tea leaves or a flavoring agent [13]. The rhizome of PS, also known as Huang Jing, is a natural product that may potentially alleviate sleep disturbances as demonstrated in previous animal models of insomnia [14,15,16], which showed no apparent adverse effects [17]. Most recently, a study revealed that the primary active component of PS rhizome is glyceryl-1-monolinoleate, a class of glycerides composed of a link between glycerol and unsaturated fatty acid via an ester bond [15,16]. Glyceryl-1-monolinoleate in PS rhizome is suggested to activate benzodiazepine receptors by binding to GABA_A_ receptors, ultimately reducing sleep latency and increasing overall sleep duration [15,16]. Specifically, preliminary test results have shown that the binding of glyceryl-1-monolinoleate to GABA_A_ receptors inhibits the binding of 3H-flumazenil, one of the major benzodiazepine antagonists [15,16]. As glyceryl-1-monolinoleate is also involved in the process of neurotransmission, investigating its sleep-enhancing properties may introduce novel ways of natural treatment for insomnia.

Considering these recent findings from animal studies, we aimed to investigate the effects of PS rhizome extract on the improvement of sleep disturbances in humans. A randomized, double-blind, placebo-controlled clinical trial that consists of a four-week administration period of either PS rhizome extract or placebo was conducted in 80 individuals with mild insomnia. The changes in sleep quality were evaluated by a series of measures including sleep diary, self-report questionnaire, and actigraphy. In addition, in order to measure the potential neurological changes induced by the administration of PS rhizome extract, we observed for changes in cerebral perfusion in regions that are known to play an important role in insomnia. Specifically, we hypothesized that a four-week administration of PS rhizome extract would improve sleep quality, as well as change the cerebral perfusion within the default mode network (DMN) since DMN changes were reported to be associated with insomnia [18,19].

## 2. Materials and Methods

### 2.1. Participants

Potentially eligible participants were recruited at Ewha Womans University. A total of 131 participants were screened for eligibility. The inclusion criteria were as follows: (1) Aged between 20 and 65 years; (2) had mild insomnia, which is defined as having one or more of the following complaints related to sleep, including difficulty falling asleep, maintaining sleep, or returning to sleep after waking up in the middle of the night, and feeling restless in the morning without any impairment or distress in daytime activities; and (3) had one or more of the abovementioned symptoms occur at least once a week for more than two consecutive months. Participants who met any of the following criteria were excluded: (1) Had primary sleep disorders (e.g., narcolepsy, sleep-related breathing disorders, periodic limb movement disorder, restless leg syndrome, and parasomnia) as determined by subjective report and medical history; (2) had taken any medications or substances that can affect sleep (e.g., benzodiazepines, corticosteroids, statins, etc.) within the past two months prior to enrollment; (3) had clinically significant impairment in social, occupational, academic, or behavioral functions attributable to sleep disturbance; (4) had significant major medical or neurological comorbidities; (5) had a lifetime history of Axis 1 psychiatric disorder, psychotic disorder, or bipolar disorder; (6) had a history of multiple adverse drug reactions or allergic reactions to PS and/or other related substances.

**Figure 1:**
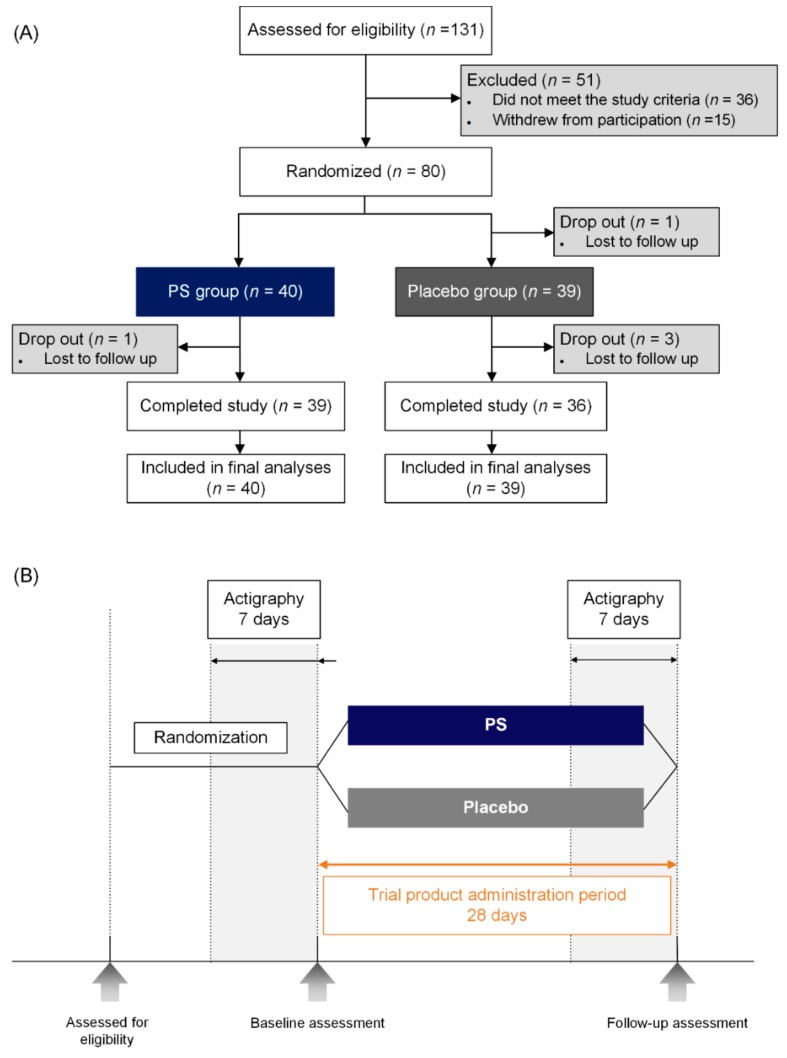
(**A**) Flowchart of the clinical trial. Eighty participants were enrolled and randomly assigned into one of two groups: 40 participants received placebo (Placebo group) and 40 received PS (PS group). (**B**) Study procedure. After randomization, the baseline evaluation was performed, which included the assessment of the Athens Insomnia Scale (AIS), objective sleep measures acquired using actigraphy, and multimodal brain MRI data. A follow-up visit was scheduled upon completion of the administration period. PS; *Polygonatum sibiricum.*

The study protocol was approved by the institutional review board of Ewha Womans University and was registered at ClinicalTrials.gov (http://clinicaltrials.gov, Identification number: NCT03337789). This clinical trial was conducted from November 2016 to December 2017, in accordance with the ethical principles of good clinical practice and the current version of the Declaration of Helsinki. All participants provided written informed consent prior to enrollment in the clinical trial. The flowchart of participants throughout the clinical trial is depicted in Figure 1A.

Enrolled participants were randomly assigned into one of two groups in a one-to-one ratio: 40 participants received placebo (Placebo group) and 40 received PS rhizome extract (PS group). The sample size of the participants were determined under the assumption that 10% of the enrolled participants would withdraw from the study, and a power of 0.80 at the 5% statistical significance level. The randomization allocation sequence was generated using a computer-generated randomization log by a third party who was not involved in the trial. All participants and investigators were blinded to the treatment assignment. Allocation concealment was also implemented since the random allocation sequence was only accessible by authorized personnel who are not involved in the conduction of this clinical trial.

### 2.2. Procedures

At baseline, sleep-related outcomes were evaluated using the Athens Insomnia Scale (AIS) and actigraphy, along with multimodal brain magnetic resonance imaging (MRI) scans (Figure 1B). Medical history and sleep history were taken, and physical examination was conducted with the measurement of vital signs and routine laboratory tests, including complete blood count, blood chemistry, and urinalysis. Upon the completion of the baseline evaluation, participants received four weeks of treatment with PS rhizome extract or placebo. Participants were instructed to take one tablet daily for four consecutive weeks, at approximately 30 min to an hour prior to going to bed. They were also instructed to refrain from taking any other medication that may affect their sleep throughout the four-week administration period. All outcome measures at baseline were re-evaluated at the follow-up visit upon completion of the four-week administration period.

The clinical trial product of PS rhizome extract was manufactured by Kolmar BNH (Chungcheong, Korea). The PS rhizome was first prepared into extract form using hot water extraction then was filtered, concentrated, and dried accordingly. All preparation steps for the PS tablet were identical to those previously reported [15,16]. Each PS rhizome extract tablet consisted of 500 mg of the prepared PS rhizome, of which the nutritional values are stated elsewhere (Supplementary Table S1), and 300 mg of miscellaneous ingredients (Supplementary Table S2). The placebo tablet was indistinguishable from the PS tablet in appearance, taste, and weight, and contained a mixture of excipient that is harmless to the human body with strict exclusion of containing any PS rhizome extract.

### 2.3. Outcome Measures

The primary outcome measure was the change in AIS score before and after the four-week administration period. The secondary outcome measures were changes in actigraphy parameters, as well as levels of cerebral perfusion within the brain regions of DMN. Cerebral perfusion was measured using the pseudocontinuous arterial spin labeling (pCASL) technique, which has been demonstrated to be reliable and reproducible across varying time intervals [20] and can provide absolute and noninvasive quantification of cerebral blood flow (CBF) in physiological units (mL/min/100 g tissue) [21].

#### 2.3.1. Athens Insomnia Scale (AIS)

The AIS is a standardized self-report questionnaire that evaluates sleep-related problems [22]. It consists of eight items that assess nocturnal sleep problems and daytime dysfunction, where higher AIS scores indicate greater severity of insomnia symptoms [22,23]. The current study used the previously validated Korean version of the AIS [24].

#### 2.3.2. Actigraphy

Actigraphy is an accelerometer that is worn on the wrist, and can measure total sleep time (TST), sleep efficacy, and minutes scored as wake after sleep onset (WASO). The Actigraph GT3X+ (Actigraph^®^, Pensacola, FL, USA) was employed to determine objective sleep quality and quantity in a naturalistic setting. The Actigraph GT3X+ has been previously validated and considered to be a reliable method in estimating sleep characteristics [25]. All sleep parameters were determined using the Actilife 6-software (Version 6.13.1, ActiGraph, Pensacola, FL, USA). Actigraphy data were acquired for two weeks as follows: One week before initiation of the clinical trial product, and during the last week of the administration (Figure 1B).

#### 2.3.3. Sleep Diaries

Daily sleep diaries were used to keep logs of self-reported sleep parameters including time of bedtime, time falling asleep, time of wake, and number of awakenings. The sleep diaries were recorded for five weeks, starting at one week before the administration period until the end of the administration period, in order to accompany the wearing of actigraphy for the data acquisition period.

#### 2.3.4. MRI Data Acquisition

All MR imaging data were acquired using a 3.0 Tesla Philips Achieva MR scanner (Philips Medical System, Best, The Netherlands) equipped with a 32-channel head coil at the Ewha Brain Institute (Seoul, Korea). Structural images were acquired using a high-resolution, high-contrast, T1-weighted three-dimensional (3D) magnetization-prepared rapid gradient echo (MPRAGE) imaging sequence with the following acquisition parameters: Echo time (TE), 3.4 ms; repetition time (TR), 7.4 ms; flip angle, 8°; field of view (FOV), 220 × 220 mm^2^; slice thickness, 1 mm; 180 contiguous sagittal slices. Perfusion imaging was performed with a balanced pCASL single-shot echo-planar imaging (EPI) sequence with the following parameters: TE, 11 ms; TR, 4000 ms; FOV, 220 × 220 mm^2^; voxel size, 2.75 × 2.75 mm^2^; 18 slices (thickness/gap = 6/0 mm); labeling duration, 1650 ms; post labeling delay, 1600 ms; number of controls/labels, 40 pairs. The labeling plane was positioned 85 mm below the center of the imaging volume, perpendicular to the internal carotid arteries. A single-shot EPI proton density (M0) image without any labeling or background suppression was acquired for each slice to measure the equilibrium longitudinal magnetization of arterial blood (TR = 6000 ms). After each session, participants reported whether they were awake during the session, and the imaging data of subjects who were asleep during the pCASL imaging scan were excluded from the analysis.

#### 2.3.5. Measurement of Cerebral Perfusion

Regional CBF was measured using the FMRIB Software Library tool (FSL, http://www.fmrib.ox.ac.uk/fsl). The equilibrium magnetization of arterial blood flow was estimated using the M0 image at voxel-level for calibration and absolute CBF quantification. The reconstructed control and label images underwent motion correction, pair-wise subtraction, then were averaged to obtain the mean perfusion-weighted image. The fitting of the kinetic model and calibration in reference to the M0 image were also performed using the Bayesian Inference for Arterial Spin Labeling (BASIL) tool (http://fsl.fmrib.ox.ac.uk/fsl/fslwiki/BASIL). The calibrated images were quantified into absolute CBF maps using the single compartment model previously described [26]. Each participant’s CBF maps were co-registered to their structural image using a linear registration tool (FLIRT), then registered to the Montreal Neurological Institute 152 (MNI152) standard space using a nonlinear registration tool (FNIRT). Registration parameters produced by the nonlinear registration process were then used to warp the perfusion images into a standard 2 × 2 × 2 mm^3^ MNI152 template. Afterward, the perfusion images were spatially smoothed with a 4.7 mm full width at half maximum (FWHM) Gaussian kernel before the statistical analysis.

#### 2.3.6. Selection of the Region of Interest for the DMN

Region of interest (ROI) masks for the DMN were generated with a threshold of *z*  =  2.33 (*p*  =  0.01), which included the bilateral medial prefrontal cortex (mPFC), precuneus, posterior cingulate cortex (PCC), and interior parietal lobule (IPL), as previously described [27]. The ROI masks for the DMN were nonlinearly registered to the subject-specific gray matter mask acquired from the individual T1-weighted image, with a threshold at 60% tissue probability (Supplementary Figure S1).

### 2.4. Clinicial Examiniation and Safety Measures

All participants underwent detailed physical and neurological examinations. Medical and substance use history including caffeine intake were acquired. Safety and tolerability of the clinical trial product were monitored and documented at every visit, including monitoring for adverse events. Routine laboratory tests including liver and renal function tests were also performed as safety measures at the baseline and follow-up assessment.

### 2.5. Compliance with Clinical Trial Product

Compliance with the clinical trial product was evaluated using a self-report questionnaire. Throughout the administration period, participants were asked to record daily whether they had administered the clinical trial product using a yes or no response, as well as the time at which they took the clinical trial product. At the end of the four-week administration period, participants were asked to return all unused products to the trial site, and the number of remaining tablets were counted to confirm the validity of the participants’ response. Individuals who took 90% or more of the assigned clinical trial products were considered compliant.

### 2.6. Statistical Analysis

For the baseline demographic data, student t-test and chi-square test or Fisher’s exact test were used to analyze continuous and dichotomous variables, respectively. All data analyses were conducted on an intent-to-treat (ITT) basis. Analyses for the primary efficacy measure were performed using a mixed model analysis for repeated measures data, where the treatment group, visit, and visit-by-group interaction were included as fixed effects, and baseline scale scores, age, and sex were included as covariates. The within-subject factor was considered a random effect. An alpha value of less than 0.05 was considered to be statistically significant based on a two-tailed test. All data were analyzed using Stata SE, version 11.0 (Stata, College Station, TX, USA).

For the actigraphy data, the difference in objective sleep measures was calculated using the abovementioned methods. For the perfusion image data, change in cerebral perfusion between post- and pre-administration of the clinical trial product was calculated by the differences in absolute perfusion value at timepoint 1 from those at timepoint 2. The group comparisons for these time-differences in perfusion images were performed according to an a priori generated ROI mask of the DMN using the Randomise tool implemented in FSL (version 2.9, http://fsl.fmrib.ox.ac.uk/fsl/fslwiki/Randomise). The results were corrected for multiple comparisons using Monte Carlo simulation with 10,000 iterations implemented in the AlphaSim utility (http://afni.nimh.nih.gov/pub/dist/doc/program_help/AlphaSim.html). A threshold derived from a combination of *p* < 0.01 and a cluster size of a minimum of 304 mm^3^ was used to correct for multiple comparisons at *p* < 0.05. Then, the values from the significant clusters were extracted for further post-hoc analyses (Supplementary Table S3).

## 3. Results

### 3.1. Demographic Characteristics

The baseline demographic and clinical characteristics did not significantly differ between groups including age, sex, and caffeine intake (Table 1). Of the 80 enrolled participants, a total of 75 participants (93.8%) completed the study (Figure 1A). The completion rates did not differ between groups, where the completion rate was 97.5% in PS group (*n* = 39) and 90.0 % in the placebo group (*n* = 36) (*p* = 0.173). There was no significant difference in the adherence to the clinical trial product as measured by the number of unused tablets returned by the participants, and the compliance rate was 91.5% for the PS group and 90.8% for the placebo group.

### 3.2. Primary Outcome

**Figure 2:**
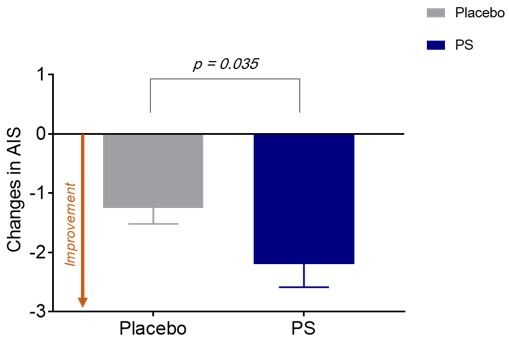
Changes in Athens Insomnia Scale after the four-week administration period. The PS group showed a significant decrease in the total AIS score compared to the placebo group over the four-week administration period (*z* = −2.1, *p* for group-by-visit interaction = 0.035). *p* < 0.05; Error bars indicate standard error. AIS, Athens Insomnia Scale; PS, *Polygonatum sibiricum.*

The PS group showed a significant decrease in total AIS score compared to the placebo group over the four-week administration period (*z* = −2.1, *p* for group-by-visit interaction = 0.035) (Figure 2).

### 3.3. Secondary Outcomes

**Figure 3:**
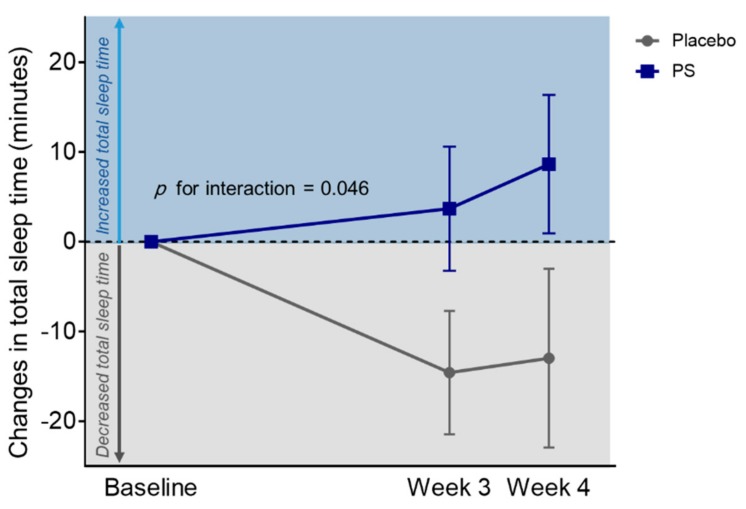
Changes in total sleep time between the PS and placebo group. Over the four-week administration period, the PS group showed significant increase in total sleep time, whereas the placebo group showed a significant decrease in total sleep time (*z* = 2.0, *p* for group-by-visit interaction = 0.046). Significant difference at *p* < 0.05; Error bars indicate standard error. PS, *Polygonatum sibiricum*.

**Figure 4:**
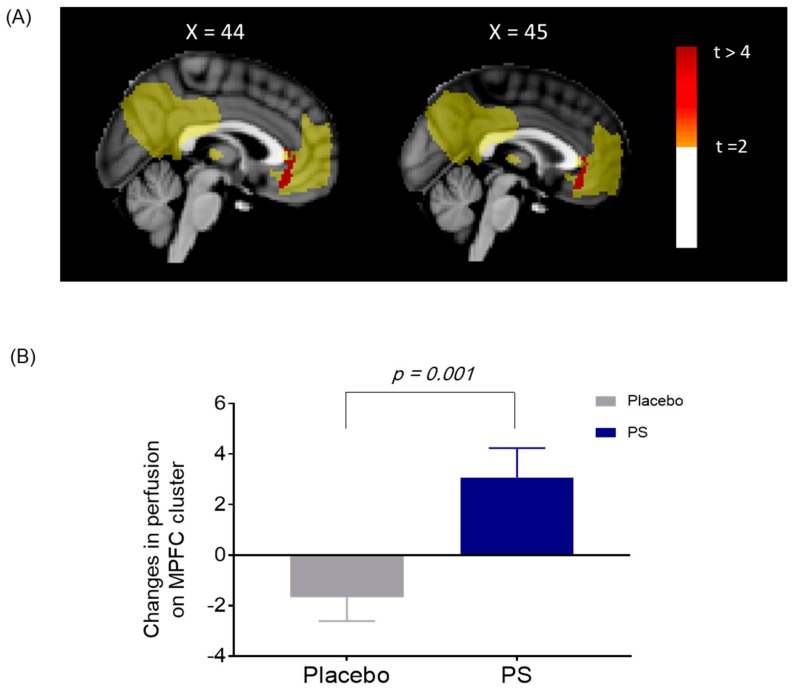
(**A**) Changes in cerebral perfusion on the default mode network (DMN) between the PS and placebo group. Significant between-group differences in changes in cerebral perfusion within the medial prefrontal cortex (mPFC) of the DMN was shown after the four-week administration period (shown in red). (**B**) Increased cerebral perfusion on the mPFC in the PS group. The values were extracted from the mPFC for post-hoc analysis, which were significant clusters as shown in Figure 4 (A). Cerebral perfusion of the mPFC increased in the PS group, while decreased in the placebo after the four-week administration (*z* = −3.0, *p* for group-by-visit interaction = 0.001). mPFC, medial prefrontal cortex; PS, *Polygonatum sibiricum*

Actigraphy data showed significant treatment group-by-visit interaction in changes in TST over the four-week administration period (*z* = 2.0, *p* for group-by-visit interaction = 0.046) (Table 2 and Figure 3). Significant treatment group-by-visit interaction was also found in changes in cerebral perfusion within the mPFC of the DMN, where cerebral perfusion increased in the PS group, while cerebral perfusion decreased in the placebo group after the four-week administration period (*z* = −3.0, *p* for group-by-visit interaction = 0.001) (Figure 4 and Table S3).

### 3.4. Correlation Analysis between Changes in Cerebral Perfusion and Sleep Parameters

There were no significant correlations between changes in cerebral perfusion and changes in AIS scores (*r* = −0.17, *p* = 0.304) or TST (*r* = −0.01, *p* = 0.951) in the PS group.

### 3.5. Safety Evaluation

None of the participants reported any adverse events, and there were no clinically relevant abnormalities as determined by the clinical assessments. Changes in vital signs, including blood pressure and pulse rate, did not differ between groups throughout the study period. All laboratory findings indicated that there were no significant differences between groups (Supplementary Table S4).

## 4. Discussion

The current study is the first randomized, double-blind, placebo-controlled clinical trial on the efficacy and safety of PS rhizome extract in relation to sleep disturbances in individuals with mild insomnia. Results of this study suggest that a four-week administration of PS rhizome could improve sleep disturbances in the form of sleep quality and sleep duration for mild insomnia. Specifically, participants in the PS group showed significant improvement in AIS score, as well as increased total sleep time, compared to the placebo group. In addition, PS administration was significantly associated with increased cerebral perfusion within the DMN, particularly the mPFC, as measured by perfusion imaging.

One of the major findings in the current study is that the PS group showed significant improvement in both subjective sleep disturbances and objective sleep quantity, which is consistent with our previous findings using animal models [15,16]. Although the exact mechanisms underlying the effects of PS rhizome on sleep promotion have not yet been clarified, a suggestive mechanism may entail the modification of protein and mRNA levels of GABA receptors in brain regions associated with sleep [15]. In particular, glyceryl-1-monolinoleate contained in PS rhizome has high binding affinity to GABA_A_ receptors [15], therefore, the four-week administration of PS rhizome may have modified the binding of GABA_A_ receptors of the DMN by refraining the binding of GABA antagonists. Given that GABA is a major inhibitory neurotransmitter in the brain and GABA_A_ receptors play a critical role in regulating sleep [28], the current findings may support the previous study that glycerol-1-monolinoleate contained in PS rhizome is a major contributing factor to the sleep promoting effects of PS [15].

Another plausible mechanism may be the anti-inflammatory effects of PS rhizome. Among the numerous active components of PS, including alkaloids, flavones, steroid saponins, and polysaccharides [29], polysaccharides are regarded as one of the most important biologically active compounds of PS rhizome [30]. Polysaccharides can significantly reduce free radical activity and enhance the activity of superoxide dismutase (SOD) and glutathione peroxidase [29]. Therefore, PS rhizome may improve sleep by enhancing the antioxidant capacity, which is also in alignment with previous studies that reported higher levels of oxidative stress markers and lower levels of antioxidant markers in primary insomnia [31]. Given the potential bidirectional association between insomnia and oxidative stress [31], increased antioxidant activities after PS rhizome administration may result in the improvement of sleep disturbances in insomnia.

The current study is also the first to apply cerebral perfusion neuroimaging as part of investigating the efficacy of PS rhizome in mild insomnia. Our results showed increased cerebral perfusion within the mPFC of the DMN in the PS group after the four-week administration, which supports the role of the DMN in the pathophysiology of insomnia [32]. Our results are in alignment with previous studies that demonstrated decreased regional cerebral metabolism in the DMN in insomnia [33], as well as the involvement of the mPFC in patients with primary insomnia [18]. Therefore, our current findings suggest a potential biological pathway of PS rhizome administration that enhances sleep in mild insomnia by increasing cerebral perfusion within the mPFC. Furthermore, PS rhizome is also suggested to have anti-atherosclerotic effects, such as protecting endothelial cells from injuries [29], which may also prevent various endothelial dysfunctions related to insomnia [34]. Therefore, continued administration of PS rhizome may enhance the anti-atherosclerotic capacity, thus contributing to increased cerebral perfusion. Future studies using endothelial cell markers are warranted to further investigate and validate the exact mechanisms that underlie PS rhizome administration and increased cerebral perfusion.

In terms of safety and tolerability, the current study reports no adverse effects from PS rhizome administration, which is in alignment with the prolonged ingestion of PS as tea leaves worldwide [13,35]. The dosage of PS rhizome in the current study design is comparable to the amount of PS rhizome contained in five to seven cups of commercially available *Polygonatum* tea, which consists of approximately 70 mg–100 mg of *Polygonatum* per cup. As such, our results further validate the safety of ingesting PS rhizome extract with a dose of 500 mg per day as a nutraceutical product and support its therapeutic potential for mild insomnia.

A dose of 500 mg PS extract per day, used in the current study, is more like a pharmacological dose rather than the nutritional one, as *Polygonatum* tea may not be consumed to that amount in countries outside of Asia. However, since the trial product is in the tablet form and can be administered once daily, it could be used more conveniently as a nutraceutical product relative to drinking multiple cups of *Polygonatum* tea.

When considering the significance of the current study findings, it is noteworthy that the current study design limits the efficacy of PS rhizome exclusively to individuals with mild insomnia, and the safety and tolerability of PS rhizome to a four-week administration period. Future studies using a longer administration period is warranted to further validate the safety of PS rhizome. In addition, follow-up evaluation after the cessation of the trial product would provide additional evidence on the long-term effects of the PS rhizome on the sleep enhancement.

In summary, the present study is the first to demonstrate the beneficial effects of a four-week administration of PS rhizome on the improvement in subjective and objective sleep in the case of insomnia. PS rhizome administration may help increase cerebral perfusion in the areas of the brain that are important factors to the pathophysiology of insomnia and other neuropsychological disorders, particularly the mPFC of the DMN. Our findings provide promising evidence for the use of PS rhizome as a natural supplement for mild insomnia, and may contribute to the development of novel phytomedicines with sleep-promoting properties.

## Figures and Tables

**Table 1:** Baseline demographic and clinical characteristics.

Characteristics	Study Group ^1^		*p*-Value
	Placebo (*n* = 39)	PS Group (*n* = 40)	
Age, mean (SD)	39.8 (13.0)	39.0 (12.5)	0.787
Sex, female, No. (%)	18 (46.2)	21 (52.5)	0.579
Years of education, mean (SD)	15.2 (1.91)	15.4 (2.59)	0.741
Socioeconomic status, No. (%)			
Upper	7 (18.0)	8 (20.0)	0.844
Upper Middle	13 (33.3)	16 (40.0)	
Middle	17 (43.6)	15 (37.5)	
Lower Middle	2 (5.1)	1 (2.5)	
Marriage, No. (%)			
Married	18 (46.2)	16 (40.0)	0.493
Never married	17 (43.6)	22 (55.0)	
Divorced, widowed, or separated	4 (10.3)	2 (5.0)	
Caffeine intake (mg) ^2^	121 (121)	95.4 (153)	0.413
Polygonatum tea intake (mL)	18.8 (42.9)	11.4 (22.8)	0.340

*p*-values were calculated using the independent *t*-test for continuous variables and chi-square test or Fisher’s exact test for categorical variables. ^1^ Data are presented as mean (SD). ^2^ Includes coffee, tea, cola drink, and chocolate. PS, *Polygonatum sibiricum*; SD, Standard deviation; No.; number.

**Table 2:** Improvement in sleep parameters as measured by actigraphy^1.^

Characteristics	Placebo		PS Group		*b* (SE)	*p* Value for Interaction ^2^
	Baseline (*n* = 39)	Week 4 (*n* = 32)	Baseline (*n* = 40)	Week 4 (*n* = 39)		
Actigraphy measurement						
Total sleep time (min)	354 (71.0)	346 (60.7)	365 (66.0)	373 (68.9)	10.5 (5.3)	0.046 *
Sleep efficiency (%)	82.2 (6.6)	82.2 (6.8)	83.7 (5.9)	83.6 (5.8)	−0.3 (0.4)	0.522
WASO (min)	68.3 (27.5)	66.8 (26.9)	61.4 (23.4)	61.9 (20.4)	2.3 (2.1)	0.275

* *p* < 0.05. ^1^ Data are presented as mean (SD). ^2^ Analyses for the primary efficacy measures of sleep-related *p* scores were performed using a mixed-effects model repeated-measures analysis. Visit was included as a fixed effect, while the within-subject factor was included as a random effect. Age and sex were included as covariates. Abbreviations: PS, *Polygonatum sibiricum*; SD, Standard deviation; AIS, Athens Insomnia Scale; WASO, wake after sleep onset; SE, standard error.

## Supplementary Materials

The following are available online at https://www.mdpi.com/2072-6643/11/8/1719/s1, Table S1: Nutritional Value of Polygonatum sibiricum Extract; Table S2: List of ingredients per each Polygonatum sibiricum tablet; Table S3: Cluster information of differences in changes of perfusion between Placebo and PS groups; Table S4: Laboratory data of serum as safety measures; Figure S1: ROI mask for default mode network in the current study.
