# Supplementary material for: Efficacy of Polygonatum sibiricum on Mild Insomnia: A Randomized Placebo-Controlled Trial
Source: Nutrients. 2019 Jul 25;11(8):1719. doi: 10.3390/nu11081719 (PMC6723095; doi:10.3390/nu11081719)
Supplement: Supplementary file 1 [file nutrients-11-01719-s001.pdf]

Supplementary Table S1. Nutritional Value of *Polygonatum sibiricum* Extract

Ingredient	Amount of ingredient per tablet
Carbohydrate	432.6 mg
Sugar	39.9 mg
Protein	50.4 mg
Lipid	0.1 mg
Fiber	31.8 mg
Ash content	16.7 mg

Supplementary Table S2. List of ingredients per each *Polygonatum sibiricum* tablet

Ingredients	Amount
Polygonatum sibiricum extract	500.0 mg
Microcrystalline cellulose	88.0 mg
Maltodextrin	40.0 mg
Powdered milk mixture	128.8 mg
Magnesium stearate	9.9 mg
Hydroxypropyl methylcellulose (HPMC)	20.8 mg
Glycerol fatty acid esters	2.1 mg
Titanium dioxide	8.0 mg
Gardenia yellow	2.4 mg
Total	800.0 mg

Supplementary Table S3. Cluster information of differences in changes of perfusion between Placebo and PS groups

Brain regions	Cluster size (mm^3)	Maximum *t* value	MNI atlas coordinate (location of maximum *t*-value)		
			x	*y*	*z*
Default Mode Network					
Medial prefrontal cortex, right	560	3.2	44	80	38

Supplementary Table S4. Laboratory data of serum as safety measures

	Placebo (*n* = 39)	PS group (*n* = 40)	All (*n* = 79)
Absolute neutrophil count (10^9/L)			
Baseline	3.29 (1.15)	3.04 (1.37)	3.16 (1.26)
Follow-up	3.32 (1.24)	3.20 (1.25)	3.25 (1.24)
Hemoglobin (g/L)			
Baseline	141 (12)	141 (13)	141 (12)
Follow-up	140 (14)	139 (13)	139 (13)
Hematocrit (Proportion of 1.0)			
Baseline	0.42 (0.04)	0.42 (0.04)	0.42 (0.04)
Follow-up	0.42 (0.04)	0.42 (0.04)	0.42 (0.04)
Platelet (10^9/L)			
Baseline	254 (54.5)	251 (69.8)	252 (62.3)
Follow-up	258 (57.4)	252 (76.4)	255 (67.6)
Serum creatinine (µmol/L)			
Baseline	72.5 (17.7)	72.5 (17.7)	72.5 (17.7)
Follow-up	70.7 (17.7)	70.7 (17.7)	70.7 (17.7)
Serum BUN (mmol/L)			
Baseline	4.68 (1.36)	4.75 (1.36)	4.71 (1.36)
Follow-up	4.50 (1.18)	4.71 (1.46)	4.61 (1.32)
AST (µkat/L)			
Baseline	0.31 (0.06)	0.32 (0.09)	0.32 (0.08)
Follow-up	0.31 (0.09)	0.33 (0.10)	0.32 (0.09)
ALT (µkat/L)			
Baseline	0.28 (0.11)	0.28 (0.13)	0.28 (0.12)
Follow-up	0.29 (0.15)	0.29 (0.16)	0.29 (0.15)
Glucose (mmol/L)			
Baseline	5.13 (0.44)	5.18 (0.48)	5.16 (0.46)
Follow-up	5.00 (0.56)	5.12 (0.69)	5.06 (0.63)
Total Cholesterol (mmol/L)			
Baseline	4.95 (0.90)	4.58 (0.81)	4.77 (0.87)
Follow-up	4.91 (0.84)	4.80 (1.40)	4.85 (1.16)
BMI (kg/m^2)			
Baseline	24.0 (3.2)	23.8 (3.2)	23.9 (3.2)

Follow-up	23.7 (3.1)	23.7 (3.4)	23.7 (3.2)

Data are presented as mean (standard deviation). PS, *polygonatum sibiricum*; BUN, blood urea nitrogen; AST, aspartate transaminase; ALT, alanine aminotransferase; BMI, body mass index

Supplementary Figure S1. ROI mask for default mode network in the current study.

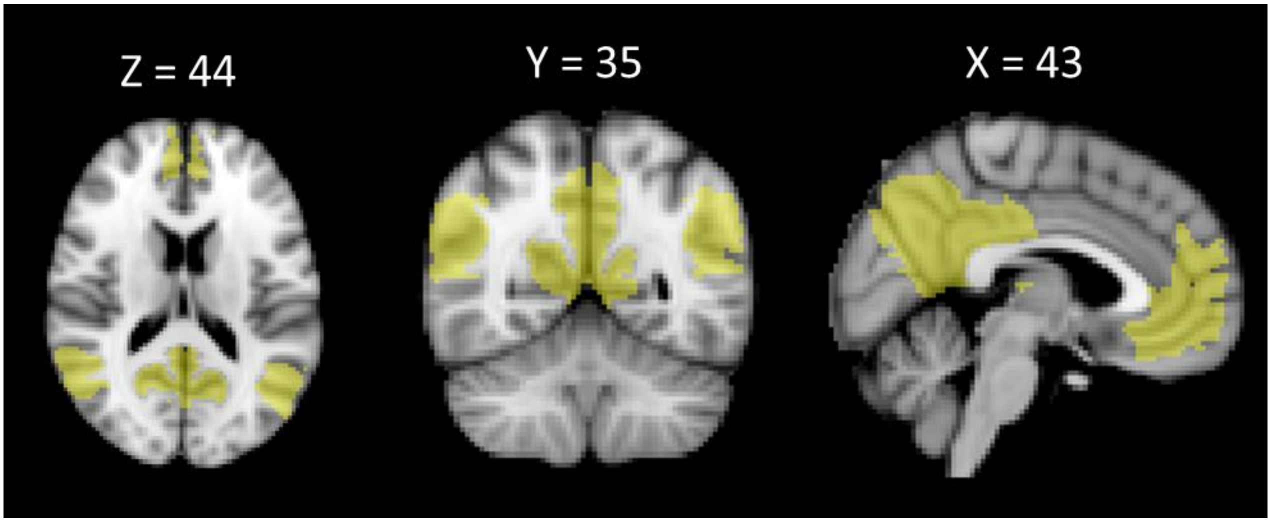

ROI masks for the DMN were generated with a threshold of $z = 2.33$ ($p = 0.01$), which included the bilateral mPFC, ACC, precuneus, PCC and IPL as described in previous research (Smith et al., 2009). The ROI masks for the DMN were non-linearly registered to the subject-specific gray matter mask from the T1-weighted images, which was thresholded at 60% tissue probability. ROI, region-of-interest; DMN, default mode network; mPFC, medial prefrontal cortex; PCC, posterior cingulate cortex; IPL, inferior parietal lobule
